# Phytochemical Constituents and Ameliorative Effect of the Essential Oil from *Annona muricata* L. Leaves in a Murine Model of Breast Cancer

**DOI:** 10.3390/molecules27061818

**Published:** 2022-03-10

**Authors:** Juan Pedro Rojas-Armas, Jorge Luis Arroyo-Acevedo, Miriam Palomino-Pacheco, José Manuel Ortiz-Sánchez, James Calva, Hugo Jesus Justil-Guerrero, Americo Castro-Luna, Norma Ramos-Cevallos, Edwin César Cieza-Macedo, Oscar Herrera-Calderon

**Affiliations:** 1 Laboratory of Pharmacology, Faculty of Medicine, Universidad Nacional Mayor de San Marcos, Av. Miguel Grau 755, Lima 15001, Peru; jprojasarmas@yahoo.com (J.P.R.-A.); jlarroyoa@gmail.com (J.L.A.-A.); hjustilg@unmsm.edu.pe (H.J.J.-G.); eciezam@unmsm.edu.pe (E.C.C.-M.); 2 Laboratory of Biochemistry, Faculty of Medicine, Universidad Nacional Mayor de San Marcos, Av. Miguel Grau 755, Lima 15001, Peru; mirianpp7@hotmail.com; 3 Laboratory of Physiology, Faculty of Medicine, Universidad Nacional Mayor de San Marcos, Av. Miguel Grau 755, Lima 15001, Peru; josemanuel4470@yahoo.es; 4 Departamento de Química, Universidad Técnica Particular de Loja, San Cayetano Alto s/n, Loja 1101608, Ecuador; jwcalva@utpl.edu.ec; 5 Research Institute for Pharmaceutical Sciences and Natural Resources, Faculty of Pharmacy and Biochemistry, Universidad Nacional Mayor de San Marcos, Jr. Puno 1002, Lima 15001, Peru; acastrol@unmsm.edu.pe (A.C.-L.); nramosc@unmsm.edu.pe (N.R.-C.); 6 Department of Pharmacology, Bromatology and Toxicology, Faculty of Pharmacy and Biochemistry, Universidad Nacional Mayor de San Marcos, Jr. Puno 1002, Lima 15001, Peru

**Keywords:** antitumor, medicinal plant, volatile oil, monoterpenes, sesquiterpenes, guanábana, soursop

## Abstract

*Annona muricata* leaves are traditionally used as an anticancer plant in the world. The aim of this study was to evaluate the ameliorative effect of the essential oil from *Annona muricata* leaves (EOAm) in an experimental model of breast cancer and to determine the volatile constituents with gas chromatography-mass spectrometry (GC-MS). Thirty female rats were assigned to five groups: the control group; the DMBA (7,12-dimethylbenz[α]anthracene) group; and three groups received daily EOAm doses of 50, 100, and 200 mg/kg/day, plus DMBA, respectively. After 13 weeks of treatment, tumors were analyzed pathologically and biochemical markers in serum were noted. As a result, in GC-MS analysis, 40 compounds were identified and 4 of them were abundant: Z-caryophyllene (40.22%), followed by α-selinene (9.94%), β-pinene (8.92%), and β-elemene (7.48%). Furthermore, EOAm in a dose-dependent form produced a reduction in tumor frequency and the accumulated tumor volume was reduced by 50% and 71% with doses of 100 and 200 mg/kg, respectively. Serum levels of reduced glutathione (GSH) increased and malondialdehyde (MDA) decreased significantly compared to the DMBA group. Serum levels of vascular endothelial growth factor (VEGF) decreased significantly from 70.75 ± 7.15 pg/mL in the DMBA group to 46.50 ± 9.00 and 34.13 ± 11.50 pg/mL in groups treated with doses of 100 and 200 mg/kg, respectively. This study concludes that the EOAm leaves showed an ameliorative effect in a murine model of breast cancer.

## 1. Introduction

Breast cancer (BC) is a cancer that is frequently diagnosed in women worldwide, leading to the second cause of death in women. Currently, BC represents a serious problem in public health in developing countries due to a high incidence and mortality [1]. In 2020, there were an estimated 276,480 new cases of BC and 42,170 deaths from this cause in the United States [2]. The protocol to treat BC depends on the tumor subtype and anatomical stage, and may include surgical resection, endocrine therapy, chemotherapy, antibodies and postoperative radiation [3]. Additionally, chemoresistance is the insensitivity of cancer cells to therapy, being an important limiting factor in the treatment of BC [4]. Due to their lack of selectivity over tumor cells and normal cells, chemotherapeutic agents, along with their beneficial effects, produce common side effects, such as myelosuppression, alopecia, diarrhea, nausea, vomiting, and stomatitis [5]. On the contrary, the average cost of treating BC is expensive and generally increases with the advanced stages of the disease [6]. This situation has triggered the screening of biomolecules from natural products with anticancer properties against BC, to be synthesized and assayed in preclinical studies, considering its better profile of efficacy, safety and lower cost. Under these conditions, natural products or medicinal plants with scientific evidence might be an alternative.

*Annona muricata* L. (Annonaceae family) is found in Africa, South America and Southeast Asia [7]. It is known by various common names according to each country: anona de puntas, sinini, graviola, soursop, sauersak, sirsak, Khan thalot, durian belanda, catuche, etc. [8]. The bark, leaves, roots, fruits and seeds of this plant are widely used in traditional medicine from different countries to treat various diseases, such as insomnia, catarrh, febrifuge (Nigeria), kidney problems, hypertension (Bolivia), galactagogue, diarrhea, arthritis (Brazil), malaria, febrifuge, inflammation (Colombia), diarrhea, lactagogue (Cuba), rheumatism (Ecuador), febrifuge, spasms, parasites (India), diabetes, gastric cancer, bronchitis, asthma (Mexico), prostate cancer, diabetes, rheumatism, arthritis (Nigeria), allergies, stomach ulcers (Panama), gastritis, diabetes, inflammation, cancer, anxiety (Peru), and malaria (Vietnam), and is also used as an analgesic, anthelmintic, abortifacient, and anticonvulsant (Cameroon) [9].

Approximately 212 phytochemical constituents have been isolated from *A. muricata*, in which the predominant main secondary metabolites are a type of natural polyketide, named acetogenins, as well as alkaloids, phenolic compounds and others [9]. Some compounds have presented antitumor activity, such as annonacin (an acetogenin), which induced growth arrest and apoptosis in estrogen receptors alpha (ERα) in MCF-7 breast cancer cells, and further attenuated MCF-7 xenograft tumor growth by inhibiting ERα expression, cyclin D1 and Bcl-2 in mice [10]. However, the essential oil from *A. muricata* leaves (EOAm) has a different composition than the leaves extract, because EOAm is composed of small molecules of monoterpenes and sesquiterpenes that volatilize into the environment. In addition, the variability in the composition of the essential oil has been observed, depending on the location, altitude, type of soil or other geographic or edaphic factors. According to the antecedents of *A. muricata* as an anticancer plant, the aim in this study was to determine the ameliorative effect of the essential oil from *A. muricata* leaves in a murine model of breast cancer. The phytochemical properties of EOAm were determined by gas chromatography-mass spectrometry (GC-MS) and the ameliorative effect on breast cancer was demonstrated by histological findings and biochemical markers, such as serum malondialdehyde (MDA), serum reduced glutathione (GSH), and serum vascular endothelial growth factor (VEGF) in female rats.

## 2. Results

### 2.1. Phytochemical Constituents of the Essential Oil from A. muricata (EOAm)

**Figure 1:**
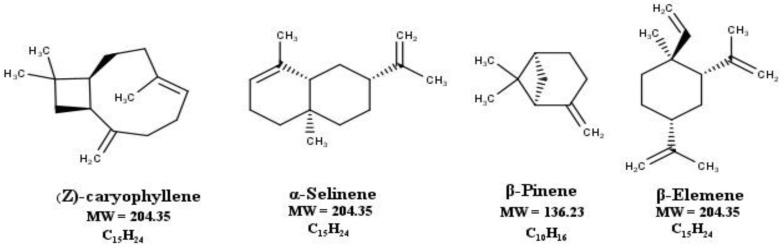
Main abundant sesquiterpene structures identified in the EOAm.

The chemical analysis revealed the presence of 40 compounds. The most abundant was Z-caryophyllene (40.22%), followed by α-selinene (9.94%), β-pinene (8.92%), and β-elemene (7.48%). Table 1 shows the total composition expressed in percentage and represents the average of three repetitions. Figure 1 reveals the main volatile components which are sesquiterpene structures determined by GC-MS.

### 2.2. Antitumor Effect of the Essential Oil of A. muricata (EOAm)

The effect of EOAm on tumor parameters is presented in Table 2. A dose-dependent reduction in the frequency of breast tumors was observed, thus 8 tumors were observed at doses of 200 mg/kg, compared to 15 tumors in the group that only received DMBA. Likewise, with the doses of 200 mg/kg, the number of animals that developed tumors decreased to 4 of the total rats in the group (4/6), which represented a 33% decrease in the incidence of tumors. This effect represents a 40% reduction in the frequency of tumors per group at doses of 100 mg/kg, while at doses of 200 mg/kg, the reduction was 47%. Tumor latency (time to appearance of tumors) was increased up to 9 days at doses of 200 mg/kg. Mean tumor volume decreased significantly (*p* < 0.05%), up to 46% at a dose of 200 mg/kg of EOAm. The cumulative tumor volume was also reduced by 50% and 71% with doses of 100 and 200 mg/kg of EOAm, respectively.

### 2.3. Histological Analysis of the Essential Oil of A. muricata (EOAm)

**Figure 2:**
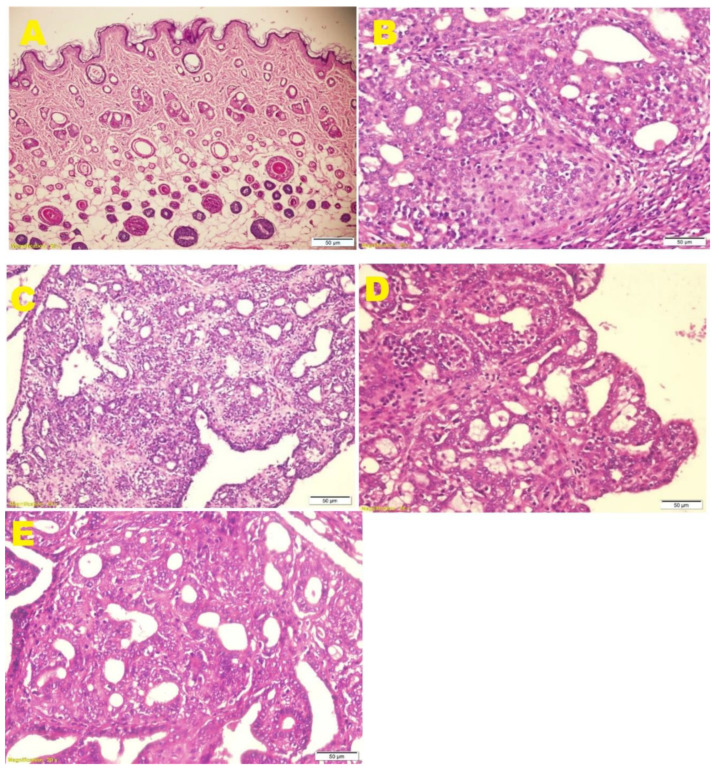
Photomicrographs of mammary cancer in rats induced by DMBA and treated with EOAm. (**A**) Control group, (**B**) DMBA, (**C**) DMBA + EOAm 50 mg/kg, (**D**) DMBA + EOAm 100 mg/kg, (**E**) DMBA + EOAm 200 mg/kg.

In the histopathological analysis, in the DMBA group, a predominantly solid pattern was observed with some tubular formations, marked nuclear pleomorphism, areas with a lymphoid inflammatory reaction, areas with necrosis, numerous mitoses and little tubular differentiation, which was classified as histological grade III. (Table 3, Figure 2B). In the groups treated with EOAm, an improvement in tubular differentiation was observed with the three dosage levels tested; however, with the doses of 50 mg/kg, a pattern with papillary areas, tubular formations and marked nuclear pleomorphism were observed (Figure 2C); whereas, with the doses of 100 and 200 mg/kg of EOAm, only moderate nuclear pleomorphism was observed compared to the marked nuclear pleomorphism of the DMBA group. Furthermore, the number of mitoses decreased, and the histological grade was I in both groups (Figure 2D,E, Table 3).

### 2.4. Biochemical Markers in Animals Treated with the Essential Oil from A. muricata (EOAm)

**Figure 3:**
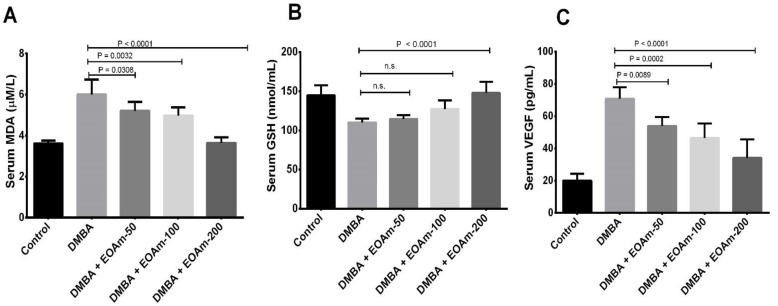
Serum levels of MDA (**A**), GSH (**B**), and VEGF (**C**) in rats treated with *A. muricata* essential oil (EOAm). Values expressed as mean ± S.D. n.s., non-significant.

In Figure 3A, serum levels of malondialdehyde (MDA) decreased significantly with doses of 200 mg/kg to 3.64 ± 0.27 µM/L compared to 6.01 ± 0.72 µM/L in the DMBA group (*p* < 0.05). In Figure 3B, serum levels of reduced glutathione (GSH) increased significantly with 200 mg/kg to 147.87 ± 14.31 nmol/mL compared to 110.34 ± 4.59 nmol/mL in the DMBA group (*p* < 0.05). Figure 3C shows serum vascular endothelial growth factor (VEGF) levels decreased significantly from 70.75 ± 7.15 pg/mL in the DMBA-induced only group to 46.50 ± 9.00 and 34.13 ± 11.50 pg/mL (*p* < 0.05) in the groups treated with EOAm at doses of 100 and 200 mg/kg, respectively.

## 3. Discussion

Based on the chemical analysis by GC-MS, this study showed similar reports from EOAm of other investigations in the world. In this study, the major component determined was Z-caryophyllene (40.22%), followed by α-selinene (9.94%), β-pinene (8.92%), and β-elemene (7.48%). In Nigeria, (E)-caryophyllene was 38.9% and eugenol was 30.2% [12]. In France, EOAm was the main metabolite in β-caryophyllene (31.4%) [13]; in Benin, the main constituents were β-caryophyllene (13.6%), followed by δ-cadinene (9.1%), epi-α-cadinol (8.4%), and α-cadinol (8.3%) [14]. Furthermore, in Vietnam, the components were β-pinene (20.6%), germacrene D (18.1%), α-pinene (9.4%), p-mentha-2,4(8)-diene (9.8%), β-elemene (9.1%), and bicycloelemene (5.8%) [15]. This indicates that the variability in EO of the chemical composition when they were analyzed by the same method is basically quantitative and these findings might be explained due to the origins of the species or geographical factors, among others.

Regarding the ameliorative effect produced by EOAm, EO produced a decreased dose-dependency on tumor incidence, tumor frequency/group, tumor volume, and increased tumor latency. Such effects could be related to the study by Owolabi, which demonstrated notable in vitro cytotoxicity on breast cancer cells (MCF-7) with 99.2% of the activity at a concentration of 100 μg/mL [12]. Likewise, the effect could also be due to the presence of EOAm components, for instance, Z-caryophyllene (syn. Z-β-caryophyllene), a bicyclic sesquiterpene, isomer (isocaryophyllene) of E-β-caryophyllene (trans-caryophyllene), and humulene (α-caryophyllene). In a study, (Z)-caryophyllene was the main component of *Croton campestris* essential oil and showed cytotoxic activity against MCF-7 and colon cancer cells HT-29 [16]. Moreso, β-caryophyllene also had an anti-proliferative effect on PA-1 and OAW 42 ovarian cancer cells, inducing cell cycle arrest in the S phase, and apoptosis was mediated by caspase-3 activation [17]. Furthermore, this produced apoptosis followed by DNA fragmentation and catalytic activity of caspase-3 in mouse lymphoma tumor cell lines BS-24-1 and Epstein–Barr MoFir virus-transformed human B lymphocytes [18]. However, together, alpha-humulene and isocaryophyllene induced antitumor activity against MCF-7 and enhanced the effect of paclitaxel on DLD-1, MCF-7and L-929 tumor cell lines [17]. Additionally, β-caryophyllene oxide suppressed the constitutive activation of STAT3 in multiple tumor cell lines, such as in myeloma, breast and prostate. It is possible that its molecular mechanism is inhibiting the proliferation, inducing apoptosis, and abrogating the invasive potential of tumor cells [18]. Furthermore, it showed a significant ability to increase the anti-proliferative effect of 5-fluorouracil and oxaliplatin on Caco-2 and SW-620 colon cancer cell lines [19]. On the other hand, β-pinene increased the antitumor effect of paclitaxel against non-small cell lung cancer cells [20]. Additionally, mixing α-selinene with aromatase p450 showed that it plays a critical role in the antagonism of aromatase p450 [21].

The histological grade, which represents the morphological evaluation of the tumor’s biological characteristics and generates crucial information about the clinical behavior of breast malignancies, represents the main indicators of breast cancer progression. Therefore, it has been incorporated into algorithms and guidelines to determine the use of adjuvant chemotherapy [22]. In the present study, EOAm decreased the histological grade from III (DMBA-induced group) to histological grade II at a dose of 50 mg/kg, whilst at 100 and 200 mg/kg, it decreased to grade I, which would indicate a significant effect on tumor development because grade I or well-differentiated tumors grow slowly, while grade III or poorly differentiated tumors grow rapidly and spread more frequently (metastasis).

On the contrary, a high concentration of reactive oxygen species (ROS) has been found in almost all cancers, due to increased metabolic activity, mitochondrial dysfunction, peroxisome activity, oncogenic activity, increased activity of oxidases, cyclooxygenases, lipoxygenases and thymidine phosphorylase [23]. Alterations in lipid peroxidation have been confirmed in breast cancer, and the main marker of oxidative stress is the level of malondialdehyde (MDA); likewise, a high level of serum MDA was found in the advanced cancer stage [24]. Reduced glutathione (GSH) is the most abundant thiol in cells, which plays an important role in antioxidant defense, acts as a free radical scavenger and detoxifying agent in cells, and is useful in a multitude of biochemical processes, such as cell proliferation, cell division and differentiation [25]. In the present study, the level of GSH increased significantly with the treatment of EOAm, while MDA decreased in the serum of female rats. Thus, this would relate to the study carried out in Ghana by Gyesi 2019 [26] that evidenced the role of EOAm with a high antioxidant capacity. Hence, the effect of EOAm on induced breast cancer in rats could be due to its antioxidant activity. Otherwise, vascular endothelial growth factor (VEGF) is a potent angiogenic cytokine that is overexpressed in breast cancer [27]. In this study, the level of VEGF decreased significantly due to the effect of the treatment with EOAm, which could indicate that a probable mechanism of antitumor action could also be inhibiting the angiogenesis process and triggering low tumor latency, tumor incidence, the average volume of tumors, and cumulative tumor volume. Regarding the toxicity of the EOAm, there exists a lack of literature to support its safety, however, in a study in which 2000 mg/kg of EOAm is administered for 14 days, mortality and neurological signs were not observed, and in the histopathological findings, hepatocyte cells showed homogenization of the cytoplasm, pyknosis or peri-lobular hepatic necrosis [28]. Although the study was used at 200 mg/kg as maximum doses, further studies as chronic studies should be carried out with additional genotoxicity studies to confirm toxicity.

## 4. Materials and Methods

### 4.1. Plant Sample Preparation

Five kilograms of fresh *A. muricata* leaves were collected from the Trujillo Market, located in La Libertad, Peru in the month of February 2019, at 34 m.a.s.l. A plant sample was transported to the Natural History Museum of the National University of San Marcos for its taxonomic identification. The constancy was assigned to Id. No. 250-USM-2019.

### 4.2. Obtention of the Essential Oil from A. muricata Leaves

The essential oil of *A. muricata* leaves was obtained with the hydrodistillation method for 2 h in a Clevenger-type apparatus [29]. Water drops were dehydrated with anhydrous Na_2_SO_4_, then it was filtered and stored in an amber glass bottle under refrigeration at 4 °C until further use.

### 4.3. Analysis of the Chemical Composition of Essential Oil

The total chemical constituents were determined using the GC-MS apparatus (7890 Gas Chromatograph and 5975C Mass Spectrometer Detector, Agilent Technologies, Santa Clara, CA, USA). During the analysis of the essential oil, 10 µL of the sample was diluted in 1 mL of dichloromethane and subsequently, 1 µL of the working test was injected in order to determine the volatile components. The GC conditions were a DB-5MS column; 30 m × 250 × 0.25 µm; the temperature ramp was set up as 50 °C as the initial constant temperature for 5 min, subsequently, a gradient of 3 °C/min until 155 °C, followed by an additional gradient of 15 °C/min until 250 °C. The last temperature was maintained constant for 2 min. The injection system operated in split mode (40:1), and helium at 1 mL/min was used as a gas carrier. Secondary metabolites identification was based on a comparison of relative retention indices (RIs) and mass spectra data with the NIST20 library data and the published literature [11]. Each RI was calculated compared with a homologous series of n-alkanes C9–C25 (C9, BHD purity 99% and C10–C25, Fluka purity 99%). The relative amount (expressed as a percentage) of each compound identified in the EO was calculated by comparing the area of the corresponding peak in the chromatogram with the total area of identified peaks. No correction factor was applied.

### 4.4. Animals

The experimental protocol was approved by the ethics committee of the Faculty of Medicine of the Universidad Nacional Mayor de San Marcos (Id. N° 0281). Animals were selected according to the inclusion criteria: female Holtzman rats weighing 160 ± 20 g in body weight. The age ranged between 6 and 8 weeks. The animals were purchased from the Bioterio of the National Institute of Health, housed in clean polypropylene cages and kept in an air-conditioned environment with a constant light/dark cycle of 12 h. Then, they were acclimatized previous to the study and with free access to water and pelleted food for rodents.

### 4.5. Induction of Breast Cancer in Rats and Experimental Design

According to the procedures of Wang & Shang [30]. 30 rats were distributed to 5 groups (*n* = 6) and treated for 13 weeks with the pharmacological drugs. The selected doses were established according to previous studies of other essential oils in breast cancer models in vivo [31,32].

- Group I received physiological saline (10 mL/kg); which is considered the control group;
- Group II received DMBA by oral administration at a single dose of 60 mg/kg of body weight, diluted in olive oil;
- Groups III (EOAm 50), IV (EOAm 100), and V (EOAm 200) received the essential oil of *A. muricata* daily in doses of 50, 100 and 200 mg/kg/day of body weight, respectively, by oral administration.

Animals were euthanized with a pentobarbital overdose (100 mg/kg; subcutaneous route) and the mammary tissues were analyzed pathologically following the evaluated parameters in experimental breast cancer [31].

### 4.6. Determination of Serum Malondialdehyde (MDA) and Serum Reduced Glutathione (GSH)

The serum MDA concentration was determined according to the Buege & Aust method [33] and serum GSH was estimated following the procedure of Brehe & Burch [34].

### 4.7. Determination of Serum Vascular Endothelial Growth Factor (VEGF)

Serum VEGF was determined with a rat VEGF ELISA kit (Sigma Chemical Co., Hamburg, Germany) [35]. The procedure was carried out following the Sigma protocol. All reagents and samples were acclimatized to room temperature. Initially, 100 μL of each standard and sample was added into appropriately coded wells. The wells were incubated for 2.5 h at room temperature with gentle shaking. The resulting solution was discarded, and wells were washed using the wash solution corresponding to the kit assay. Next, 100 µL of the antibody VEFG was added to each well. The wells were incubated for 1 h at room temperature with gentle shaking. Then, it was washed and 100 µL of prepared streptavidin solution was added to each well. The wells were covered and incubated for 45 min with gentle shaking. Finally, 100 µL of TMB (3,3′,5,5′-tetramethylbenzidine) was added to each well and incubated for 30 min at room temperature. As a final step, 50 µL of the stop solution was added to each well and read at 450 nm in a microplate reader.

### 4.8. Statistical Analysis

SPSS software ver. 20.0 (IBM Corporation, NY, USA) was used to analyze data from experimental groups. Data were expressed as percentages, means and standard deviation. For the antitumor effect and the evaluated biochemical markers (MDA, GSH and VEGF), a one-way analysis of variance (ANOVA), followed by Tukey’s test were used, considering *p*-values less than 0.05 as significant.

## 5. Conclusions

In conclusion, according to the results obtained under the experimental conditions, the essential oil of *A. muricata* leaves showed an ameliorative effect in an experimental model of breast cancer in female rats. The essential oil revealed four main sesquiterpenes, such as Z-caryophyllene, α-selinene, β-pinene, and β-elemene, which were determined by GC-MS and the most abundant in the total composition. Regarding the evaluated biochemical markers, the essential oil at 200 mg/kg reduced MDA and VEGF and increased GSH. However, there was no difference between the doses of 100 and 200 mg/kg in the histopathological evaluation but improved several indicators compared with those animals with breast cancer.

## Figures and Tables

**Table 1:** Phytochemical constituents of the essential oil of *A. muricata* leaves determined by GC-MS.

Peak	Rt	Chemical Constituents	Peak Area	LRI^exp^	LRI^ref^
1	6.66	Tricyclene	3.31	926	926
2	7.29	α-Fenchene	0.69	943	945
3	8.40	β-Pinene	8.92	973	974
4	8.91	Myrcene	0.60	987	988
5	10.45	ρ-Cymene	0.23	1023	1020
6	10.65	Limonene	1.52	1027	1024
7	12.00	γ-Terpinene	0.49	1056	1054
8	14.66	cis-Rose oxide	0.71	1113	1106
9	16.62	Menthone	0.91	1154	1148
10	17.80	Santalone	0.26	1178	1173
11	20.48	Pulegone	0.93	1234	1233
12	22.71	(E)-Anethole	0.70	1281	1282
13	24.70	neoiso-Verbanol acetate	0.35	1327	1328
14	24.86	cis-Piperitol acetate	0.41	1330	1332
15	26.91	β-Bourbonene	1.27	1375	1387
16	27.23	β-Elemene	7.48	1382	1389
17	27.81	β-Longipinene	1.00	1394	1400
18	27.92	Longifolene	0.34	1402	1407
19	28.47	(Z)-Caryophyllene	40.22	1414	1408
20	29.08	α-trans-Bergamotene	0.33	1428	1432
21	29.20	Aromadendrene	1.11	1431	1439
22	29.92	α-Humulene	2.72	1447	1452
23	30.09	allo-Aromadendrene	0.86	1451	1458
24	30.76	β-Acoradiene	0.92	1466	1469
25	30.98	Germacrene D	1.71	1471	1480
26	31.29	γ-Himachalene	1.19	1479	1481
27	31.40	cis-Eudesma-6,11-diene	1.00	1481	1489
28	31.60	α-Selinene	9.94	1486	1498
29	32.04	Germacrene A	1.30	1501	1508
30	32.54	δ-Amorphene	0.42	1513	1511
31	32.91	(Z)-γ-Bisabolene	0.50	1522	1514
32	34.34	(E)-Nerolidol	1.33	1557	1561
33	34.81	Spathulenol	0.97	1568	1577
34	34.97	Caryophyllene oxide	0.71	1572	1582
35	35.13	Thujopsan-2-α-ol	1.23	1576	1586
36	35.46	Globulol	0.30	1584	1590
37	38.43	(6Z)-Pentadecen-2-one	1.59	1664	1667
38	38.58	Caryophyllene <14-hydroxy-9-epi-(E)->	0.42	1668	1668
39	38.74	n-Tetradecanol	0.39	1672	1671
40	39.63	n-Heptadecane	0.71	1695	1700
		Total components	100.00		

Rt, retention time; LRI^exp^, linear retention index calculated against n-alkanes C9–C24; LRI^ref^, linear retention index obtained from the literature [11].

**Table 2:** Histological findings in female rats treated with the essential oil from *A. muricata* leaves.

Parameters/Groups	DMBA	DMBA + EOAm50	DMBA + EOAm100	DMBA + EOAm200
Total number of tumors	15.00	12.00	9.00	8.00
Animals with tumors/Total animals	6/6	6/6	5/6	4/6
Frequency of tumors by group	2.50 ± 0.34	2.00 ± 0.26 (−20%)	1.50 ± 0.43 (−40%)	1.33 ± 0.49 (−47%)
Tumor latency (days)	66.17 ± 2.70	65.83 ± 2.31 (−0.4 days)	69.40 ± 1.69 (+3 days)	74.75 ± 1.49 (+8.6 days)
Tumor incidence (%)	100.00	100.00	83.33 (−17%)	66.67 (−33%)
Average volume of tumors (cm^3^)	0.39 ± 0.02	0.38 ± 0.02 (−3%)	0.33 ± 0.03 (−15%)	0.21 ± 0.04 * (−46%)
Cumulative tumor volume (cm^3^)	5.89	4.55 (−23%)	2.93 (−50%)	1.68 (−71%)

Values expressed as mean ± SEM. EOAm; essential oil of *Annona muricata*. * Significant difference from the DMBA group (*p* < 0.05). One-way ANOVA followed by a post hoc Tukey test.

**Table 3:** Histological grade of DMBA-induced breast cancer in rats treated with essential oil from *A. muricata* (EOAm).

Parameter/Group	DMBA	DMBA + EOAm 50	DMBA + EOAm 100	DMBA + EOAm 200
Tubular differentiation	3	2	2	2
Nuclear pleomorphism	3	3	2	2
Number of mitoses	2	1	1	1
Score	8	6	5	5
Histological grade	III	II	I	I

Histological grade according to Mod Elston & Ellis. Histopathology 1991. Grade I: 3–5, Grade II: 6–7, Grade III: 8–9. Parameter scores: tubular differentiation: 2 = 10–75%, 3 = <10%; nuclear pleomorphism: 3 = marked, 2 = moderate; number of mitoses: 1 = <7 mitoses, 2 = 7–13.

## Data Availability

All data are available in this publication.

## Sample Availability

Samples of the compounds are available from the authors.
